# Clinical and paraclinical features of small fiber neuropathy in Sjögren’s syndrome

**DOI:** 10.1007/s00415-022-11431-1

**Published:** 2022-11-04

**Authors:** Tabea Seeliger, Henrike Neelke Dreyer, Janna Margaretha Siemer, Lena Bönig, Stefan Gingele, Maike Franziska Dohrn, Nils Prenzler, Diana Ernst, Torsten Witte, Thomas Skripuletz

**Affiliations:** 1 Department of Neurology, Hannover Medical School, Carl-Neuberg-Str. 1, 30625 Hannover, Germany; 2 Department of Neurology, RWTH Aachen University, Aachen, Germany; 3 Department of Otolaryngology, Hannover Medical School, Hannover, Germany; 4 Department of Rheumatology and Immunology, Hannover Medical School, Hannover, Germany

**Keywords:** Small fiber neuropathy, Neuro-Sjögren, Sjögren’s syndrome, Skin biopsy

## Abstract

Sjögren’s syndrome is a potentially treatable cause of Small Fiber Neuropathy (SFN)—a condition that severely affects patients’ quality of life. We therefore aimed to characterize patients with SFN and Sjögren’s syndrome to raise awareness of this disease and facilitate its early recognition as an essential step for appropriate treatment. In 97 SFN patients (median age 48 years, 77% female), we studied the clinical features associated with Sjögren’s syndrome compared to the idiopathic SFN subtype. According to the current ACR/EULAR classification criteria (Shiboski et al., Ann Rheum Dis 76:9–16, 2017), 24/97 individuals (25%, median age 48.5 years, 75% female) were diagnosed with Sjögren’s syndrome. We did not observe any differences in SFN-defining sensory plus symptoms. Furthermore, intraepidermal nerve fiber densities (IENFD) were significantly lower in patients with SFN and Sjögren’s syndrome (mean 2.6 ± 1.2/mm) compared to patients with idiopathic SFN (mean 3.2 ± 1.5/mm; *p* = 0.048). There were no significant group differences when analyzing cerebrospinal fluid (CSF) parameters. We conclude that Sjögren’s syndrome-associated SFN is difficult to distinguish from idiopathic forms based on initial clinical symptoms and CSF results. However, lower IENFD values in patients with Sjögren's syndrome-associated SFN might indicate a distinct different pathomechanism in this entity compared to idiopathic SFN.

## Introduction

Sjögren’s syndrome is an autoimmune inflammation of the salivary and lachrymal glands characterized primarily by sicca symptoms of the eyes and mouth. Neurological involvement of Sjögren’s syndrome is an increasingly recognized complication [2–7]. Recent work has shown that the course of disease can be very severe but is also well treatable when effective immunosuppressive therapy is implemented: therapeutic options for severe disease courses currently include azathioprine, intravenous corticosteroids, and immunoglobulins, as well as rituximab, while other immunosuppressants can also represent reasonable choices of treatment [5, 8, 9]. Hereby, the therapeutic regimen must always be tailored to affected patients’ individual disease course. Clinically, neuropathy is the most common neurological involvement in Sjögren’s syndrome, ranging from small fiber to large fiber neuropathy [10, 11]. Interestingly, there appear to be different clinical phenotypes in patients with Sjögren’s syndrome depending on whether neurologic involvement is evident or not [12]. In patients with chronic inflammatory demyelinating polyneuropathy, an underlying Sjögren’s syndrome has been shown to be more common in females and when obvious cranial nerve impairment occurs [13].

The term small fiber neuropathy (SFN) comprises a heterogeneous disease group, the underlying origin of which remains “idiopathic” in about 50% of cases [14]. Overall, the main symptom of SFN is neuropathic pain leading to a significant daily life impairment in affected patients [15].

Although SFN is an established and frequent complication of Sjögren’s syndrome that severely affects patients’ quality of life [16, 17], knowledge of clinical features to distinguish between patients with and without underlying Sjögren’s syndrome or even to predict evident SFN in patients with Sjögren’s syndrome remains scarce [18, 19]. This is especially important, as Sjögren’s syndrome represents a potentially treatable cause of SFN [9]. We, therefore, aimed to clinically characterize patients with SFN and Sjögren’s syndrome to raise awareness and facilitate early disease recognition.

## Methods

### Study design

**Fig. 1:**
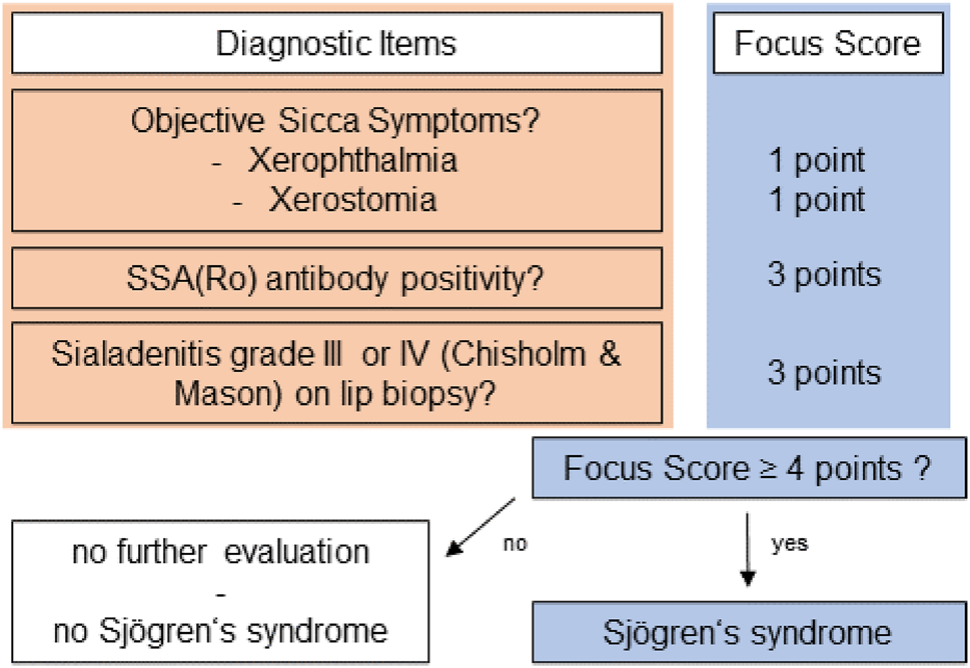
Diagnostic algorithm of Sjögren’s syndrome in clinical routine and according to Shiboski et al. [1]. Diagnosis of Small Fiber Neuropathy

We retrospectively studied the clinical features of patients with SFN and histopathologically detectable diminished intraepidermal nerve fiber density (IENFD) treated at our university hospital. Evaluation for Sjögren’s syndrome was conducted according to the current American College of Rheumatology/European League Against Rheumatism classification criteria for Sjögren’s syndrome [1, 8] as implemented into our clinical routine. The diagnostic algorithm is displayed in Fig. 1. Data analysis included the initial clinical presentation, nerve conduction studies, histopathological analysis of a skin biopsy specimen, and laboratory analysis of blood serum samples and cerebrospinal fluid (CSF). Then, the cohort was evaluated for differences between patients with SFN and Sjögren’s syndrome versus patients with SFN without Sjögren’s syndrome (idiopathic). Patients with amyloid deposits on thioflavin-S-staining were not included in the study. The local ethics committee approved the study (8172_BO_K_2018).

SFN diagnosis was prompted following the Diagnostic Criteria for SFN by Devigili et al. [10] and The Analgesic, Anesthetic, and Addiction Clinical Trial Translations, Innovations, Opportunities and Networks (ACTTION) criteria [20]. Therefore, patients were only included if they presented with clinical signs of small fiber impairment in areas suggestive for peripheral neuropathy (length- or non-length-dependent). Patients were excluded, if large fiber involvement was evident on nerve conduction studies. Skin punch biopsies were obtained according to the current guidelines of the European Federation of Neurological Societies/Peripheral nerve Society [21]. The collected specimens were frozen and sectioned (cryostat) and subsequently stained with HE and thioflavin S. In addition, immunohistochemical reaction to PGP9.5 was assessed by fluorescence microscopy for four sections. IENFD was measured and compared with normative values established by Lauria et al. [22]. IENFD values that were below the 5^th^ percentile of the proposed normative value for the respective age and sex were considered abnormal [23].

### Statistical analysis

Results were checked for normal distribution via Shapiro–Wilk-test if applicable. Differences between the groups were evaluated using Chi^2^-test for binary data and two-sided *t* test (parametric)/Wilcoxon test (nonparametric) for metric variables when applicable. *p* values ≤ 0.05 were considered significant. Statistical analysis was performed using STATA^®^ 16.1 Texas, USA. Graphs were created by R Studio 2022.02.3 © 2009–2022 RStudio, PBC and Inkscape 1.1.2 © 1989, 1991 Free Software Foundation, Inc. Boston, MA, USA.

## Results

### Patient characteristics

One hundred and twenty-four patients with evidently diminished nerve fiber density on skin punch biopsy and clinically suggestive symptoms for SFN were screened for idiopathic SFN. Patients were not included because of additionally evident diabetes mellitus in 6 cases, vitamin b12-deficiency in 5 cases, paraproteinemia in 2 cases and abnormalities on nerve conduction studies in 14 cases. Subsequently, 97 patients (median age 48 years, 77% female) treated at our university hospital between 03/2014 and 04/2022 were included in the analysis. Evaluation for possible Sjögren's syndrome according to the current ACR/ EULAR classification criteria [1] had led to the diagnosis of additional Sjögren’s syndrome in 24 patients (median age 48.5 years, 75% female). However, most patients were not found to have additional Sjögren’s syndrome and were further considered to suffer from idiopathic SFN (73/97 [75%], median 49 years, 78% female). The full baseline information is detailed in Table 1.

**Table 1:** Baseline information

	Idiopathic SFN	Sjögren’s syndrome with SFN	*p* value
*N* (%)	73 (75%)	24 (25%)	n/a
Female	57 (78%)	18 (75%)	0.8
Age at time of skin biopsy, median (IQR) (years)	49 (38–56)	48.5 (44–58)	0.2
ESSDAI, median (IQR)	n/a	6 (5–10)	n/a
ESSPRI, median (IQR)	n/a	6.7 (5.8–7.4)	n/a
Xerophthalmia	28 (38%)	17 (71%)	0.006
Xerostomia	24 (34%)	14 (61%)	0.02
Anti-SSA(Ro)-antibody positivity	0 (0%)	11 (48%)	< 0.001
Sialadenitis grade 3 or 4 (Chisholm and Mason) [24]	0/43 (0%)	18/21 (86%)	n/a
Focus Score, median (IQR)	1 (0–2)	5 (4–5)	< 0.0001

### Clinical presentation at symptom onset

Analysis of symptoms reported at the initial presentation did not reveal significant differences between groups for any of the symptoms, especially paresthesia, numbness and pain. The full analysis of both subgroups is shown in Table 2. Also, there was no significant difference concerning the duration from symptom onset to the timepoint of skin biopsy obtainment (*p* = 0.9).

**Table 2:** Symptoms reported at symptom onset and symptom duration

	Idiopathic SFN	Sjögren’s syndrome with SFN	*p* value
Symptoms reported at symptom onset, *n* (%)			
Paraesthesia	54 (77%)	16 (67%)	0.31
Pain	31 (44%)	14 (58%)	0.24
Numbness	28 (40%)	5 (21%)	0.09
Stocking/glove-like symptom distribution	12 (17%)	8 (33%)	0.09
Single-limb affection	3 (4%)	3 (13%)	0.16
Two-limb affection	33 (45%)	9 (38%)	0.51
Three-limb affection	2 (3%)	0 (0%)	n/a
All-limb affection	28 (38%)	11 (46%)	0.52
Symmetrical limb involvement	52 (71%)	15 (65%)	0.58
Facial involvement of sensory symptoms	14 (21%)	4 (17%)	0.63
Duration of symptoms			
Time from symptom onset to skin biopsy, median (IQR) (years)	1 (1–3.5)	1 (1–6.5)	0.9

### Histopathological findings

**Fig. 2:**
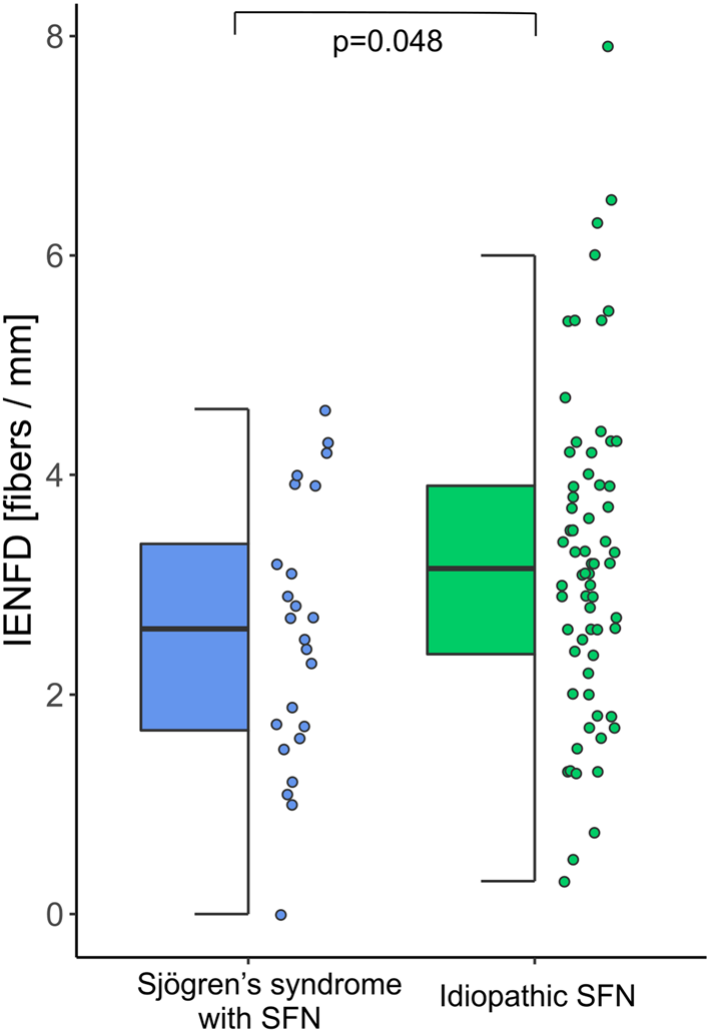
Intraepidermal nerve fiber density for Sjögren’s syndrome with SFN (blue) and idiopathic SFN (green)

Intraepidermal nerve fiber density was significantly lower in patients with SFN and Sjögren’s syndrome (mean 2.6 ± 1.2/mm) compared to patients with idiopathic SFN (mean 3.2 ± 1.5/mm; *p* = 0.048) (Fig. 2).

The frequency of subepidermal nerve plexus rarefication was similar in patients with additional Sjögren’s syndrome (10 [42%]) and in patients with idiopathic SFN (36 [49%], *p* = 0.79).

### Cerebrospinal fluid analysis

There were no significant group differences in the analysis of CSF-parameters. The full workup is shown in Table 3.

**Table 3:** Analysis of cerebrospinal fluid parameters

	Idiopathic SFN	Sjögren’s syndrome with SFN	*p* value
Cerebrospinal fluid analysis			
Cell count, median (IQR) (cells/µl)	2 (2–2)	2 (1–2)	0.18
CSF total protein, median (IQR) (mg/l)	398 (318–489)	471 (350–555)	0.24
CSF total protein elevated, *n* (%)	10/57 (18%)	6/19 (32%)	0.19
CSF Q-albumin, median (IQR)	5.4 (3.9–7.4)	6.6 (4–7.9)	0.26
CSF Q-albumin elevated	14/58 (24%)	8/19 (42%)	0.13
Evidence of oligoclonal bands	7/53 (13%)	3/17 (17%)	0.72
CSF lactate, median (IQR)	1.5 (1.4–1.7)	1.6 (1.5–1.8)	0.16

## Discussion

The quality of life of patients with SFN is severely compromised, while treatment options remain symptomatic in most cases—unless a treatable underlying condition can be identified. Sjögren’s syndrome represents such an underlying disease associated with SFN [10], with patients often responding well to treatment if appropriate immunosuppressive treatment is implemented early in the course of the disease [9]. Therefore, it is necessary to raise awareness of this entity and facilitate early detection of the disease.

### Lower nerve fiber density in SFN with underlying Sjögren’s syndrome

In this analysis, nerve fiber density in the intraepidermal skin layer was significantly lower in SFN patients with Sjögren’s syndrome than in idiopathic SFN patients. This finding suggests a greater extent of nerve destruction in patients with Sjögren’s syndrome as a possible causative systemic autoimmune disease, especially since neuropathic motor involvement can develop severe disease courses in this entity [8]. Nevertheless, motor involvement in Sjögren’s syndrome is associated with destruction of large fibers, whereas SFN manifests as neuronal damage to Aδ and C-fibers. Hence, the disease courses of the two entities may not be adequately comparable because different pathomechanisms may be involved. Hypothetically, delayed performance of skin biopsies in patients with Sjögren’s syndrome due to overlapping symptoms of this systemic disease could explain the advanced neuronal damage. However, we found no significant group difference for the duration from symptom onset to conduction of the biopsy in our cohort. Interestingly, previous studies rather showed similar nerve fiber density values in SFN patients with idiopathic, glucose-tolerance, and diabetes mellitus-associated background [25]. Therefore, the significant reduction in IENFD in patients with an underlying Sjögren’s syndrome is especially notable because the extent of IENFD reduction was not previously known to differ between causative conditions of SFN. On the other hand, the pathophysiological interpretation of the reduced nerve fibers in skin biopsies remains notional. Previous studies showed that lower values of nerve fiber density in skin biopsy are associated with reduced quality of life [26], although a correlation with the degree of neuropathic pain remains controversial [27–29]. Overall, the lower rate of intraepidermal nerve fibers in patients with SFN and Sjögren’s syndrome supports the hypothesis that SFN in Sjögren’s syndrome as an autoimmune disease is associated with more extensive nerve fiber damage than SFN without Sjögren’s syndrome.

### Consequences for the clinical routine

Practicing neurologists are frequently confronted with patients suffering from unspecific sensory symptoms while diagnostic procedures are often not only time-consuming but also frustratingly unspecific, especially when nerve conduction studies remain normal. Pure SFN is an important differential diagnosis in these cases, as affected patients may have normal nerve conduction studies. Patients usually report neuropathic pain that may present in a spontaneous and/or evoked manner and mostly with an intensity of > 4 on the visual analog scale (VAS). As a result, these patients are often severely impaired in their quality of life, which is why it is important that we focus on identifying potentially treatable conditions as early as possible in the search for the correct diagnosis. According to the diagnostic criteria for SFN [10], there are still about 10% of patients with SFN who show normal results on skin biopsy despite pathological findings on quantitative sensory testing (QST), which is why Devigili et al. suggested that the diagnosis should be prompted when at least two of the following items are abnormal: (1) clinical signs of small fiber impairment (loss of pinprick and thermosensory sensation and/or allodynia and/or hyperalgesia) with a distribution consistent with peripheral neuropathy; (2) abnormal heat and/or cooling threshold at the foot assessed by QST; (3) reduced IENF density on the distal leg. The authors also suggested that patients should be rigorously excluded from the diagnosis of pure SFN if any of the following findings were present: (1) any sign of large fiber impairment (light touch and/or vibratory and/or proprioceptive sensory loss and/or absent deep tendon reflexes); (2) any sign of motor impairment (muscle atrophy and/or weakness); (3) any abnormality on the sensorimotor NCS. The latter exclusion criteria lead to the frequent problem in clinical routine that patients who meet the diagnostic “inclusion criteria” for SFN, but at the same time have minor abnormalities on the NCS, cannot be diagnosed with SFN. This problem was also reflected in our recruitment process, as 14 patients were not included due to large fiber involvement on the NCS leading to a potential selection bias.

Analysis of clinical symptoms at disease onset revealed that the initially reported symptoms of patients with SFN do not differ between idiopathic SFN and SFN associated with Sjögren’s syndrome. This symptom overlap might also be amplified through autonomic involvement of SFN which can lead to dry eyes and mouth symptoms [30, 31]. Also, neither assessment of CSF parameters such as cell count, total protein, Q-albumin, lactate, nor evaluation for oligoclonal bands was helpful in distinguishing the groups. Therefore, clinical and paraclinical prediction of an underlying Sjögren’s syndrome in SFN patients remains a challenge.

### Limitations

Naturally, the impact of this study is limited due to its retrospective design and lack of QST data. Nevertheless, even though QST provides a reliable and objective assessment of small fiber functionality, its diagnostic yield has been shown to be lower (46.5%) than that of clinical examination and skin biopsy (diagnostic efficiency of 88.4%) [10]. One of the main previously published studies was conducted by Birnbaum et al.: patients with Sjögren’s syndrome were prospectively included and skin punch biopsies were performed at the distal leg and the proximal thigh. Data analysis showed that patients with SFN-associated neuropathy were rather male, less often SSA(Ro)-antibody positive and more frequently displayed an axonal nerve damage pattern. Nevertheless, other than the current study the work by Birnbaum et al. focused on differences between Sjögren’s syndrome with and without SFN and furthermore included patients with mixed small and large fiber involvement. Therefore, the results are difficult to translate and further studies with a prospective design and additional assessment of QST in these patients are needed to confirm our results.

Additionally, the hypothesized causative connection between Sjögren’s syndrome and SFN remains notional, even though many previous studies have described an association between the two entities [10, 11] and although we present a comparatively large cohort of patients with Sjögren’s syndrome and SFN with this study.

## Conclusion

Our results show that it is not possible to find evidence of Sjögren's syndrome in patients with SFN based on initial clinical symptoms and CSF diagnostic results. Skin biopsy results indicate more severe damage with evidence of lower IENFD levels in patients with Sjögren's syndrome associated SFN compared with idiopathic SFN.

## Data Availability

All data are available upon reasonable request.
